# The complete mitochondrial genome of an Asian crested ibis *Nipponia nippon* (Pelecaniformes, Threskiornithidae) from South Korea

**DOI:** 10.1080/23802359.2019.1680321

**Published:** 2019-10-21

**Authors:** Gyeongmin Kim, Kyu Cheol Jeong, Eun Hwa Choi, Shi Hyun Ryu, Young Jin Lim, Jumin Jun, Young-Sup Lee, Ui Wook Hwang

**Affiliations:** a School of Life Science, Graduate School, Kyungpook National University, Daegu, Republic of Korea;; b Department of Biology Education, Teachers College and Institute for Phylogenomics and Evolution, Kyungpook National University, Daegu, Republic of Korea;; c Freshwater Biodiversity Research Division, Nakdonggang National Institute of Biological Resources, Sangju, Republic of Korea;; d Animal Resources Division, National Institute of Biological Resources, Incheon, Republic of Korea;; e Institute for Korean Herb-Bio Convergence Promotion, Kyungpook National University, Daegu, Republic of Korea

**Keywords:** *Nipponia nippon*, Threskiornithidae, mitochondrial genome, Asian crested ibis, phylogenetic analysis

## Abstract

The complete mitochondrial genome sequence from the Asian crested ibis, *Nipponia nippon* (Aves, Pelecaniformes, Threskiornithidae), was determined and characterized in detail. This mitochondrial genome is 16,813 bp long, and consists of 13 PCGs, 22 tRNAs, and 2 rRNAs. The nucleotide composition is slightly biased with A + T contents of 53.79% (A, T, C, and G was 30.37%, 23.42%, 31.99%, and 14.22%, respectively). 11 PCGs are initiated by ATN codons, except for *cox1* and *cox2* with GTG instead. The phylogenetic relationships based on the maximum-likelihood and Bayesian methods showed that the placement of *N. nippon* within the order Pelecaniformes, with forming the monoclade of the family Threskiornithidae.

The Asian crested ibis, *Nipponia nippon* (Pelecaniformes, Threskiornithidae), is one of the world’s most endangered species, which is listed as Endangered on the IUCN Red List. The family Threskiornithidae including *N. nippon* were formerly known as the member of the order Ciconiiformes, and are now reclassified under the order Pelecaniformes. The Asian crested ibis was widely distributed throughout East Asian region encompassing China, Japan, Korea, and Russia in the past. In Korean Peninsula, it is known that the wild population of *N. nippon* had gone extinct by 1970s for the several reasons.

In present study, the mitochondrial genome of *N. nippon* individual from South Korean captive population (The crested ibis restoration centre, Changnyeong-gun, 35°32′46.61″N, 128°24′49.96″E) which is inbreeded by the policy for Korean conservation system, was completely sequenced and compared with the other avian mitochondrial genomes. Total genomic DNA was extracted from blood sample, deposited in Kyungpook National University from South Korea (voucher specimen no. NN201200M). The complete mitochondrial genome was amplified using PCR with 15 pairs of primer designed. The data of genome was analysed using by Clustal X version 2.1 (Larkin et al. 2007) and the phylogenetic tree was reconstructed by using IQ-Tree (Trifinopoulos et al. 2016) and MrBayes 3.2 (Ronquist et al. 2012).

The complete mitochondrial genome of *N. nippon* from Korea was 16,813 bp in length (GenBank accession no. MN047457). A total of 37 mitochondrial genes were identified: 13 protein-coding genes (PCGs), 22 tRNA genes, and two rRNA genes. *Nad6* and eight tRNAs of *trnQ, trnA, trnN, trnC, trnY, trnS(UCN), trnP, trnE* were located on the light strand, while the remaining genes were located on the heavy strand. The overall base composition of the mitochondrial genome of *N. nippon* was 30.37% for A, 23.42% for T, 31.99% for C, and 14.22% for G. *Nad3* started with ATC codon, *cox1* and *cox2* with GTG, and the remaining genes with ATG. All of the tRNA genes were capable of forming typical cloverleaf secondary structures, with an exception of *trnS(AGY)* having a lacked DHU arm in *N. nippon*. In comparison of the mitochondrial genome sequences of Korean and Japanese *N. nippon*, their similarity among 13 PCGs was the 99.92% in total. The lowest similarity was shown in the control region (89.12%).

The phylogenetic tree was reconstructed with 12 PCGs from the *N. nippon* mitochondrial genome except for *nad*6 (Figure 1). The results supported the placement of *N. nippon* within the order Pelecaniformes, with forming the monoclade of the family Threskiornithidae. Because of the unexpected phylogenetic position of *Pelecanus conspicillatus,* Pelecaniformes did not form a monophyletic group. Zhang et al. (2012) suggested a coincident viewpoint using mitochondrial genomes. Moreover, Hackett et al. (2008) found that Ciconiiformes and Pelecaniformes were intermixed in a clade based on 19 individual nucleic loci DNA sequence. It is likely that mitochondrial genome data of Korean *N. nippon* is helpful for enhancing our comprehension of the phylogeny and evolution of the orders Pelecaniformes and Ciconiiformes, and for establishing national conservation plan for an endangered bird species.

**Figure 1.**
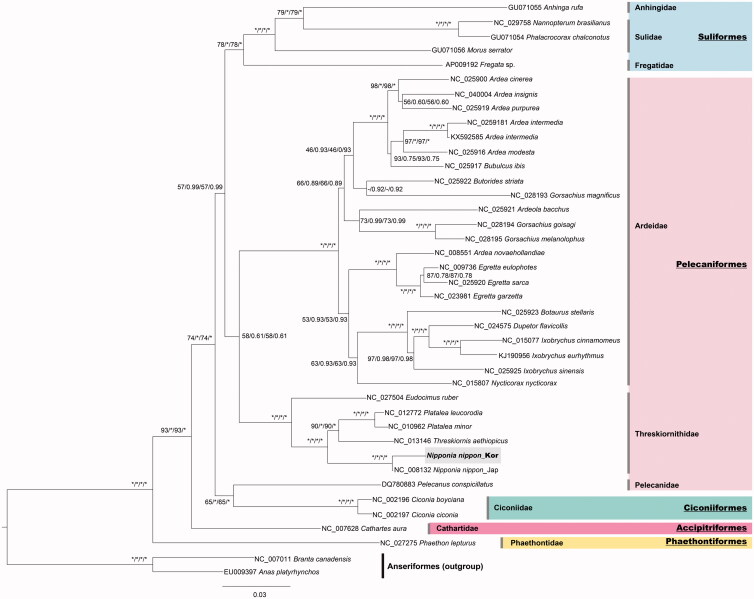
Bayesian inference tree of 37 water birds and 1 land bird inferred from 12 PCGs (except for the *nad6* gene) showing phylogenetic relationships among the four orders, eight families and phylogenetic position of *N. nippon* (Korea). Phylogenetic relationships among 37 water birds and 1 land bird based on the four following concatenated data sets: (1) Bootstrapping value of a maximum likelihood tree based on nucleotide sequence alignment, (2) Bayesian posterior probability based on nucleic acid sequence alignment, (3) bootstrapping value of a maximum likelihood tree based on amino acid sequence alignment, and (4) Bayesian posterior probability based on amino acid sequence alignment. An asterisk on a branch indicates that it was supported by 100% bootstrap value (BP) and a Bayesian posterior probability of 1.00.
